# Supplementary material for: Aging Activates the Immune System and Alters the Regenerative Capacity in the Zebrafish Heart
Source: Cells. 2022 Jan 20;11(3):345. doi: 10.3390/cells11030345 (PMC8834511; doi:10.3390/cells11030345)
Supplement: Supplementary file 1 [file cells-11-00345-s001.zip › Supplement_Reuter_et_al(Cells)/Supporting_Information.pdf]

Supporting material -

Aging activates the immune system and alters the regenerative capacity in the zebrafish heart

Running title: Aging of the zebrafish heart

Authors:

Hanna Reuter [1], Birgit Perner [1,2], Florian Wahl [1], Luise Rohde [1], Philipp Koch [3], Marco Groth [4], Katrin Buder [5], Christoph Englert [1,6,+]

Affiliation:

[1] Molecular Genetics Laboratory, Leibniz Institute on Aging-Fritz Lipmann Institute (FLI), Jena, Germany.

[2] Core Facility Imaging, [3] Core Facility Life Science Computing, [4] Core Facility DNA Sequencing, [5] Core Service Histology/Pathology/EM, Leibniz Institute on Aging-Fritz Lipmann Institute (FLI), Jena, Germany.

[6] Institute of Biochemistry and Biophysics, Friedrich-Schiller-University Jena, Jena, Germany.

+ Corresponding author: Christoph.Englert@leibniz-fli.de

Supporting material

Figure Legends Supplement

Figure S1

Figure S2

Figure S3

Figure S4

Figure S5

Movie legends

Table legends

Supplementary experimental procedures

References

Supporting material

Figure S1 – related to Figure 1. Validation of differentially expressed genes in old vs. young ventricles

(A) Expression as log2 of normalized counts of the two DEGs *ncf1* and *enpp1*. n=3 **(B)** qRT-PCR analysis of the two genes *ncf1* and *enpp1* (from A) in young (6 months), middle old (1.5 years) and old (4 years) ventricles confirms up-regulation in old. n=4. Standard deviation is indicated. *ncf1*: one-way ANOVA p=0.02998; post-hoc Tukey HSD p_{adj}(6months-4years)= 0.0263137; *enpp1:* one-way ANOVA p=0.01050; post-hoc Tukey HSD p_{adj}(4years-1.5years)= 0.0277478, p_{adj}(6months-4years)= 0.0131282.

Figure S2 – related to Figure 3. Cellular senescence in the fish heart. Expression of complement component *c6* is associated to ventricle areas close to atrium and bulbus arteriosus.

(A) Genes annotated with the GO:0090398 term for cellular senescence in zebrafish as well as the senescence-associated genes *cdkn1a* (p21) and *cdkn2a/b* (p16) are highlighted (red dots). FC: fold change, p_{adj} of 0.05 is indicated as a dashed red line. **(B)** Senescence-activated beta-galactosidase staining of heart sections reveals an increased presence of senescence cells in the old zebrafish heart. **(C)** Expression of the complement component gene *c6* as log2 of normalized counts in young and old ventricle. n=3 **(D)** *In situ* hybridization on heart sections reveals *c6* expression in in the ventricle (V) close to the transition towards bulbus arteriosus (BA) or atrium (A) and associated to pericardial fat (PF). The sense control serves as a negative control. Dotted boxes indicate areas of inset views (to the right). All scale bars are 100 µm. young: 5 months; old: 41 months. **(E)** Oil red O staining of heart sections reveals pericardial fat droplets in young (8 months) and old (36 months) fish. Boxes indicate areas of inset views (below). Scale bars are 200 µm in overviews and 50 µm in insets. **(F)** Fluorescent *in situ* hybridization for *c6* (red) on heart sections in combination with immunostainings against the pan leukocyte marker L-Plastin (white) reveal immune cells not as the main source for *c6* expression. Boxes indicate areas of respective inset views (below). White arrows point towards *c6*$^{-}$/L-Plastin^{+}, orange arrows towards *c6*$^{+}$/L-Plastin^{-} and yellow arrows to *c6*$^{+}$/L-Plastin^{+} cells. Scale bar in overview represents 50 µm and 10 µm in insets.

Figure S3 – related to Figure 4. Immune cells are of myeloid origin and accumulate in ventricles upon aging close to vessels and pericardial fat.

(A) Analysis strategy to identify enriched immune cell populations. Unique markers of 10 immune cell populations were identified from single cell RNA sequencing data of zebrafish whole kidney marrow (Tang, Iyer et al. 2017) and compared to the 745 up-regulated DEGs (from Figure 1C). **(B)** Normalized percentages of unique marker genes among the 745 genes indicates macrophage markers as most enriched in old ventricles. **(C)** Whole-mount immunostaining for GFP (green) of old transgenic *mpeg:YFP* hearts reveals mpeg+ cells in the area of pericardial fat (yellow arrows) and away from fat droplets (red arrow). 7.2 µm section visualized by lightsheet microscopy. **(D)** Whole-mount immunostaining for Spi1b (red) stains coronary vessels of the fish heart in addition to nuclei of Spi1b-psotive cells (inset view).

Lightsheet microscopy and 3D projection. Box indicates area of inset view. Scale bar of inset view is 50 µm. **(E)** Whole-mount immunostaining of a *pu.1:EGFP* reporter line with anti-Spi1b and anti-GFP reveal an overlap of Spi1b-positive nuclei and GFP positive cells. Scale bar is 20 µm. **(F)** Whole-mount immunostaining for the pan-leukocyte marker L-Plastin (green) and Spi1b (red) reveals L-Plastin+ cell accumulation in old fish next to coronary vessels (yellow arrow) and in areas with no vessels (white arrow). Lightsheet microscopy and 3D projection.

Figure S4 – related to Figure 5. Mpeg+ cell counts are increased in old.
Segments counted (mpeg+ cells) at all 90 time points of *ex vivo* imaging are increased in numbers in old compared to young. A reporter line expressing *YFP* under the control of the *mpeg1.1* promoter was used. n=540 images (6 biological replicates per age at 90 time points), mean(young)=161.6, mean(old)=223.6, Welch two-sample t- test $p < 2.2e{-16}$, age young: 3x 6 months and 3x 12 months, age old: 3x 4 years 7 months and 3x 5 years.

Figure S5 – related to Figure 6. Regeneration response and collagen accumulation is changed in old cryo-injured or sham treated hearts, respectively.
(A) Whole-mount immunostaining of old (4 years 1 month) and young (1 year 2 months) fish hearts 30 days post cryoinjury for the muscle marker Tropomyosin (red), Fibronectin (green) and the pan-leukocyte marker L-Plastin (white) reveal increased wound (dotted line) size in old. Lightsheet microscopy and 3D projection. Scale bars are 100 µm. **(B)** Quantification of collagen in uninjured young (8 months) and old (37 months) ventricle. n(young)=6; n(old)=6; Wilcoxon rank sum test p=0.0260. **(C)** Quantification of collagen in old (3 years 10 months) and young (1 year 4 months) ventricle at 30 dpi (from E). n(young)=3; n(old)=6; wo-sample t- test p=0.0018; control=sham treated. **(D)** AFOG staining of sections of control (sham treated) young and old hearts at 30 dpi. Example zoom-in views of the ventricular border regions are shown, arrows indicate regions of collagen accumulation. Scale bars are 50 µm.

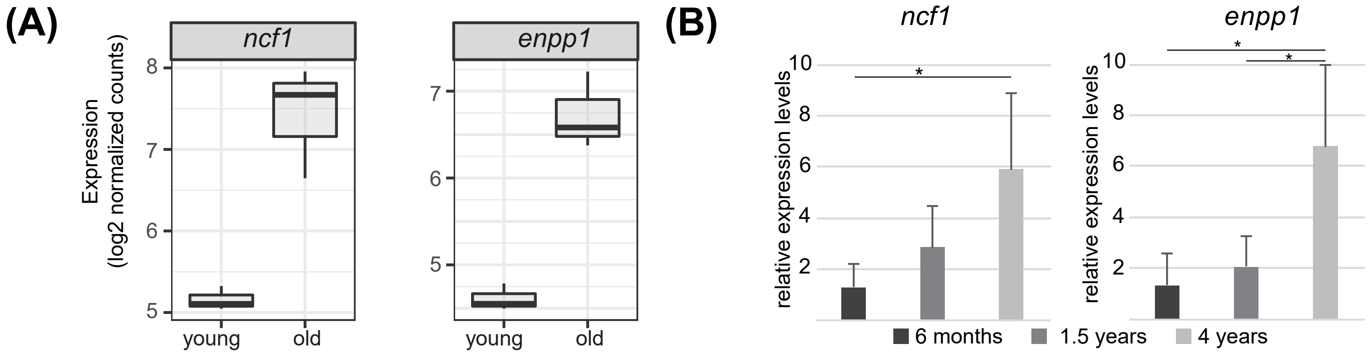

(A)
ncf1
enpp1
Expression
(log2 normalized counts)
8
7
6
5
young
old
(B)
ncf1
enpp1
relative expression levels
10
8
6
4
2
*
6 months
1.5 years
4 years

Figure S1

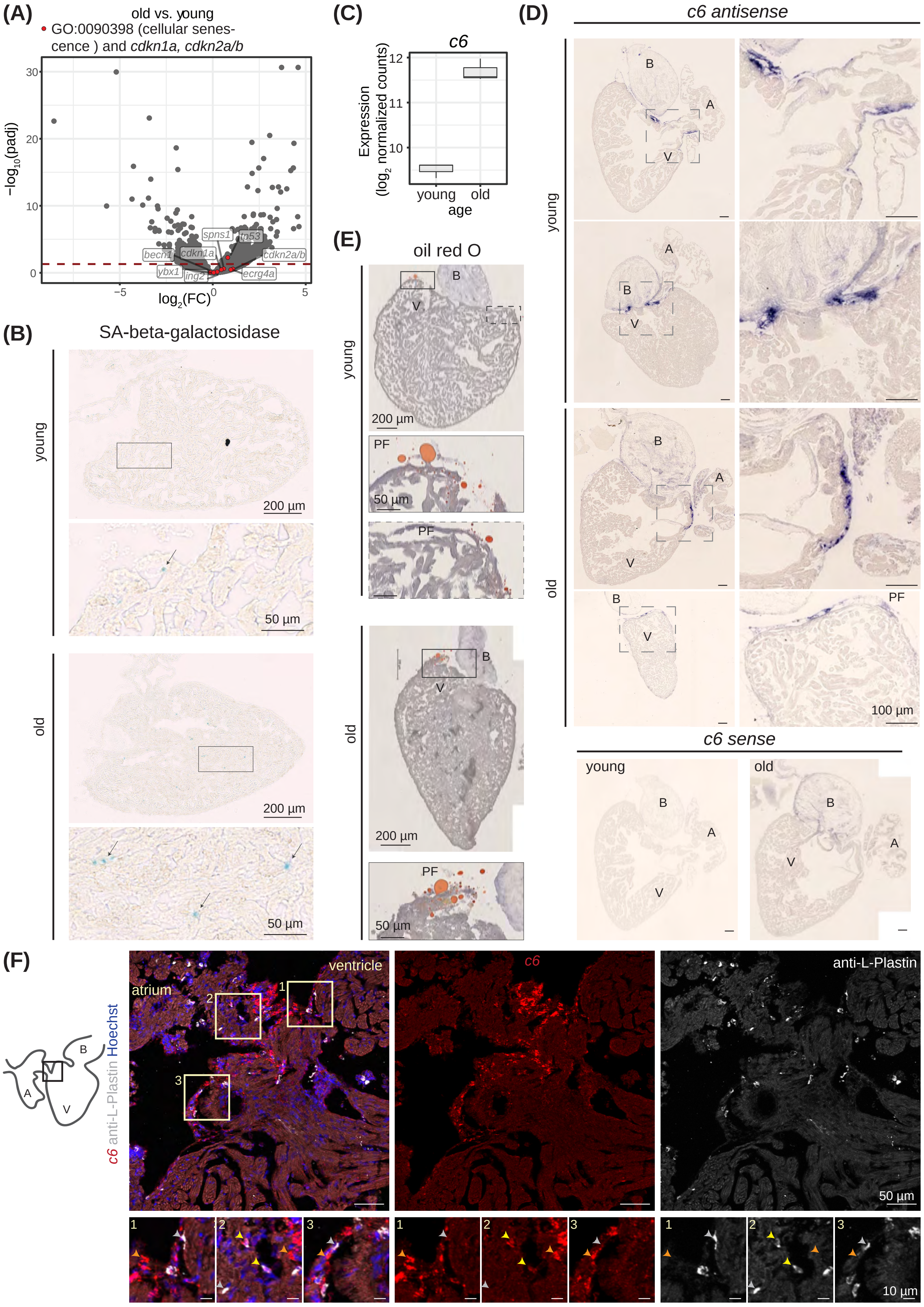
(A)
old vs. young
GO:0090398 (cellular senes-cence) and cdkn1a, cdkn2a/b
−log10(padj)
log2(FC)
spns1
tp53
becn1
cdkn1a
cdkn2a/b
vbx1
ing2
ecrg4a
(B)
SA-beta-galactosidase
young
old
200 µm
50 µm
(C)
c6
Expression (log2 normalized counts)
young
old
age
(D)
c6 antisense
young
old
B
A
V
PF
100 µm
c6 sense
(E)
oil red O
PF
200 µm
50 µm
(F)
atrium
ventricle
c6
anti-L-Plastin
Hoechst
50 µm
10 µm

Figure S2

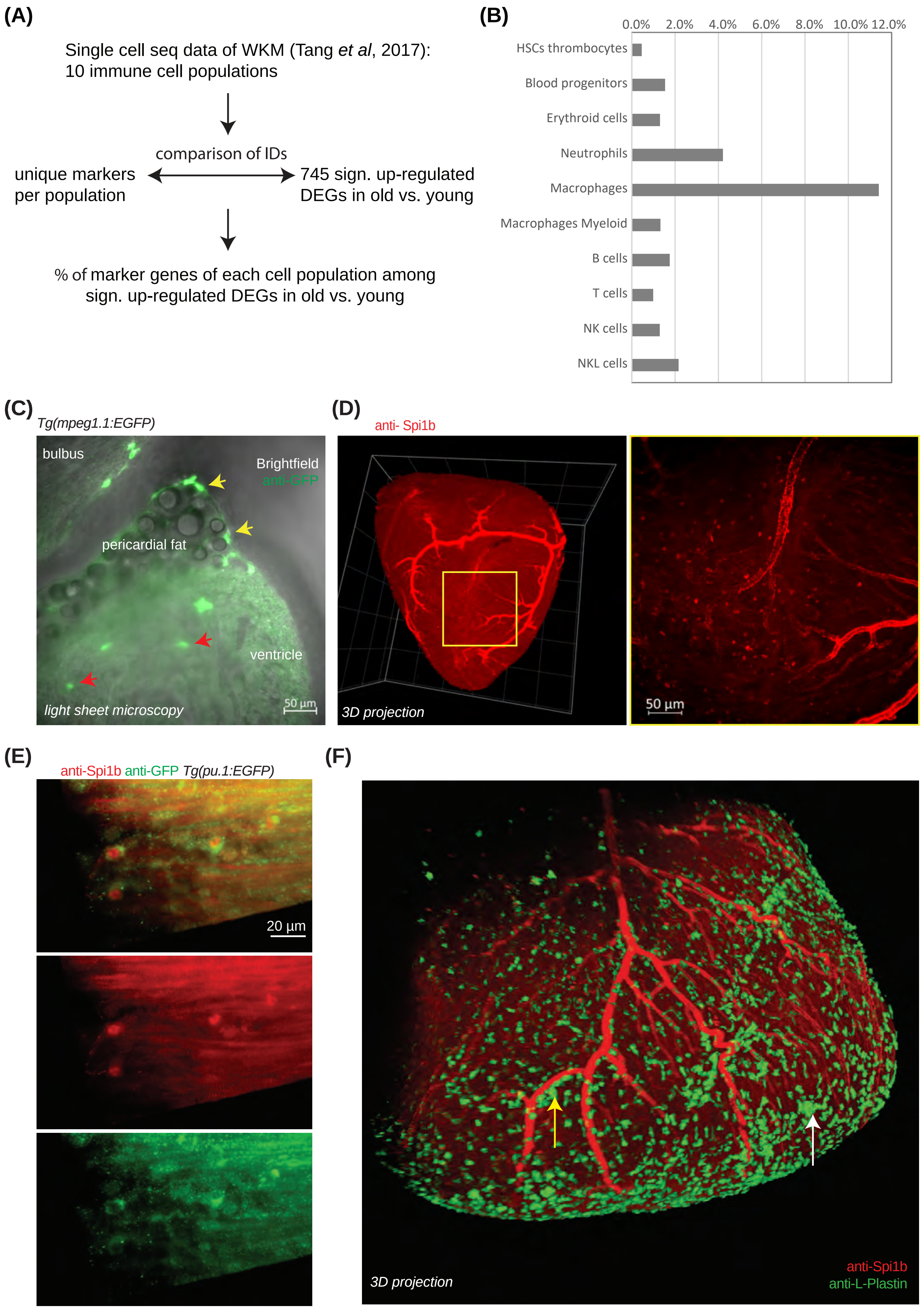
(A)
Single cell seq data of WKM (Tang et al, 2017):
10 immune cell populations
comparison of IDs
unique markers
per population
745 sign. up-regulated
DEGs in old vs. young
% of marker genes of each cell population among
sign. up-regulated DEGs in old vs. young
(B)
0.0% 2.0% 4.0% 6.0% 8.0% 10.0% 12.0%
HSCs thrombocytes
Blood progenitors
Erythroid cells
Neutrophils
Macrophages
Macrophages Myeloid
B cells
T cells
NK cells
NKL cells
(C)
Tg(mpeg1.1:EGFP)
bulbus
Brightfield
anti-GFP
pericardial fat
ventricle
light sheet microscopy
50 µm
(D)
anti- Spi1b
3D projection
50 µm
(E)
anti-Spi1b anti-GFP Tg(pu.1:EGFP)
20 µm
(F)
3D projection
anti-Spi1b
anti-L-Plastin

Figure S3

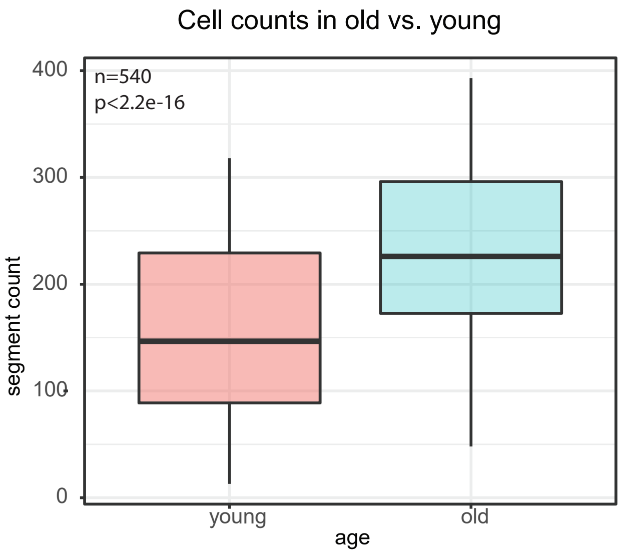
Cell counts in old vs. young
n=540
p<2.2e-16
400
300
200
100
0
segment count
young
old
age

Figure S4

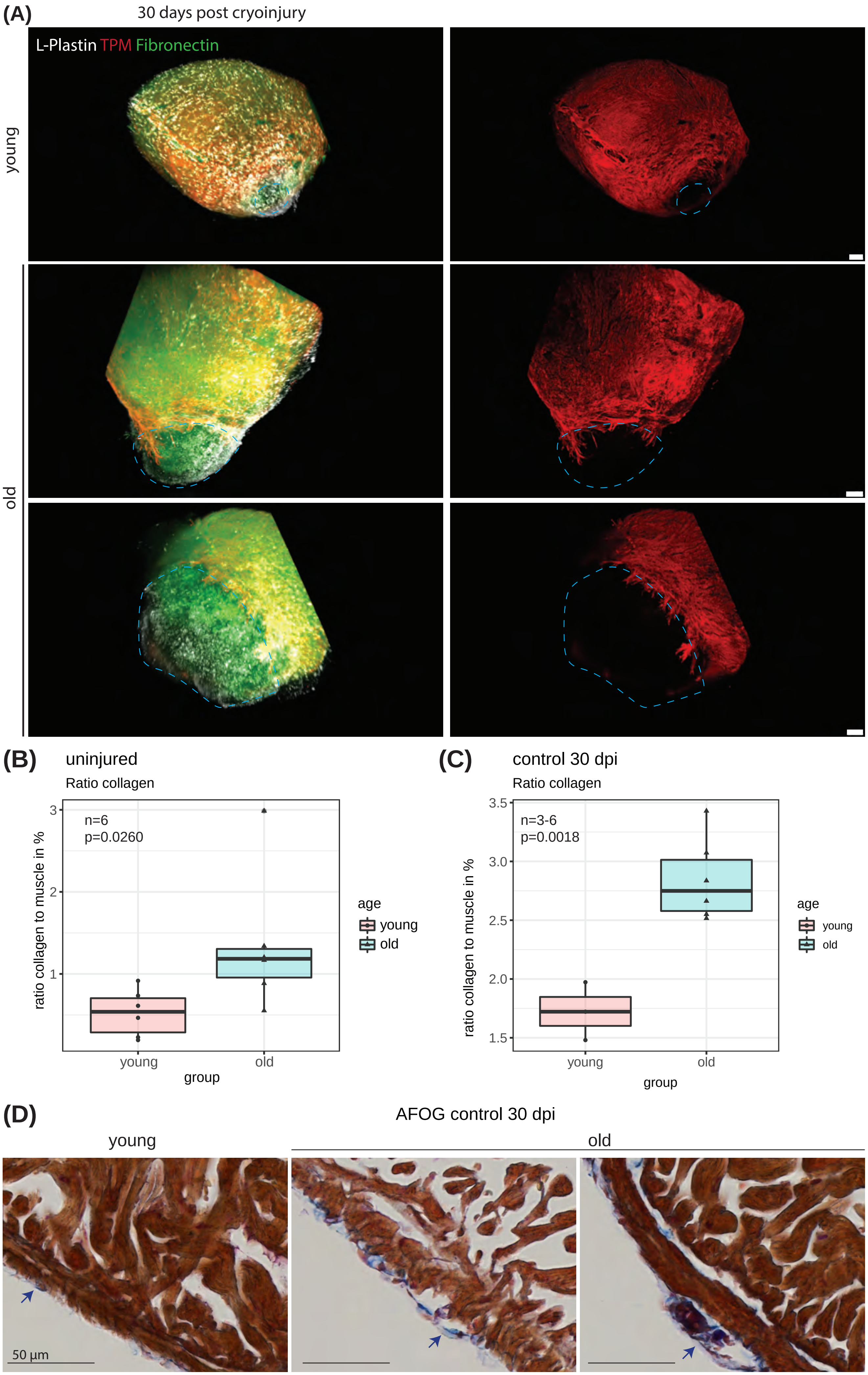
(A)
30 days post cryoinjury
L-Plastin TPM Fibronectin
young
old
(B)
uninjured
Ratio collagen
ratio collagen to muscle in %
n=6
p=0.0260
3
2
1
young
old
group
age
young
old
(C)
control 30 dpi
Ratio collagen
ratio collagen to muscle in %
n=3-6
p=0.0018
3.5
3.0
2.5
2.0
1.5
young
old
group
age
young
old
(D)
AFOG control 30 dpi
young
old
50 µm

Figure S5

Supplemental movie legends

Movie 1: Movie along z of whole-mount immunostaining of a young (1 year) fish heart. Pan-leukocyte marker L-Plastin in green and the muscle marker Tropomyosin in red reveal resident immune cells in the heart ventricle. Lightsheet microscopy. Scale bar is 20 µm.

Movie 2: Movie along z of whole-mount immunostaining of a old (4 years) fish heart. Pan-leukocyte marker L-Plastin in green and the muscle marker Tropomyosin in red reveal increased resident immune cells in the heart ventricle with roundish shape. Lightsheet microscopy. Scale bar is 20 µm.

Movie 3: *Ex vivo* live imaging of a young (6 months) zebrafish heart by lightsheet microscopy, using a reporter line expressing YFP under the control of the *mpeg1.1* promoter, reveals random movement of resident macrophages. Images were acquired every 2 min for 90 cycles (2 hours 58 mins).

Movie 4: *Ex vivo* live imaging of an old (5 years)zebrafish heart by lightsheet microscopy, using a reporter line expressing YFP under the control of the *mpeg1.1* promoter, reveals random movement of resident macrophages . Images were acquired every 2 min for 90 cycles (2 hours 58 mins).

Supplemental table legends

Table S1: All genes analyzed during transcriptome profiling of old and young zebrafish ventricles including genes that are differentially expressed (DEGs) in the comparison of old vs young (related to Figure 1B).

Table S2: 520 Ensembl IDs of DEGs of old vs. young ventricles in overlap with DEGs in hearts of *klotho* mutant vs. wildtype (related to Figure 1C, D).

Table S3: Results of GO enrichment analysis of the GO term category “Biological Process” for down regulated DEGs (related to Figure 2A).

Table S4: Results of GO enrichment analysis of the GO term category “Cellular Compartment” for down regulated DEGs (related to Figure 2B).

Table S5: Results of GO enrichment analysis of the GO term category “Biological Process” for up regulated DEGs (related to Figure 3A).

Table S6: Results of GO enrichment analysis of the GO term category “Biological Process” for 520 DEGs from Table S2.

Supplemental experimental procedures

qRT-PCR

Total RNA was isolated from ventricles using trizol according to the manufacturer's protocol. RNA was reverse transcribed with iScript cDNA synthesis kit (Bio-Rad) and qRT-PCR was carried out in triplicates for each sample using SYBR GreenER (Thermo Fisher Scientific) on the CFX384 Real-Time System (Bio-Rad). PCR efficiencies and relative expression were calculated according to (Pfaffl 2001). For normalization, we used *ef1α*. Primers used*: enpp1* forward: CCGCTGCAGTGATAAAGTCA, *enpp1* reverse: CAGGTGCCTTCATTTACGCA, *ncf1* forward: TGTCAGCTCATTCGGGACTT, *ncf1* reverse: CAGGTGCCTTCATTTACGCA, *ef1α* forward: AAGAGAACCATCGAGAAGTTCGA, *ef1α* reverse: ACCCAGGCGTACTTGAAGGA. Statistics (one-way ANOVA, post-hoc Tukey HSD) were computed with R.

Data comparison with immune cell populations

We identified unique markers of 10 immune cell populations published as single cell RNA sequencing data of the whole kidney marrow (Tang, Iyer et al. 2017) using R by filtering for exclusively upregulated genes in each of the 10 immune cell populations. We thencompared the Ensembl IDs of these unique markers to Ensembl IDs of the 745 up-regulated DEGs identified in this study.

In situ hybridization on tissue sections

Digoxigenin labeled *In situ* probes were *in vitro* transcribed from PCR products generated with primers containing overhangs for T7/SP6 promoter (DIG RNA Labeling, Roche). Primers used:
c6 forward atttaggtgacactatagaaACCTGGTCCCAATGCACTAA,
c6 reverse: taatacgactcactatagggCCCTCCTAATGTCCCAGAGC.
In situ hybridizations on 10 μm paraffin sections were performed as described previously (Leimeister, Bach et al. 1998). Anti-Dig-AP antibody (Roche) was used at 1:5000 and color reaction performed with BM-Purple (Roche). For fluorescent *in situ* hybridizations, anti-Dig-POD antibody (Roche) was used at 1:100 and labeling performed using tyramide signal amplification with Opal 570 (1:100) (Akoya Biosciences, Perkin Elmer). For immunofluorescence staining after fluorescent *in situ* hybridizations, samples were further blocked in 10% goat serum, 2% BSA, 0.2% Tween in PBS, followed by incubation with primary antibody in blocking solution: chicken anti-L-Plastin at 1:500 (kind gift of P. Martin). Secondary anti-chick antibody was used at 1:500 (Alexa Fluor secondary antibodies, Thermo Fisher Scientific). DNA was counterstained with Hoechst. Slides were mounted with Aqua Polymount (PolyScience).

Senescence-activated (SA) β-galactosidase staining

SA-β -galactosidase staining was performed on cryo tissue sections similar as described for whole-body staining (Kishi, Uchiyama et al. 2003). Briefly, slides were rinsed four times with 1x PBS (pH=6.0) and incubated at 37 °C for 18-20 h with fresh SA-β-gal staining solution (5 mM potassium ferricyanide, 5 mM

potassium ferrocyanide, 2 mM MgCl2 in PBS at pH 6.0, 1 mg/ml X-GAL (dissolved in DMSO)). Reaction was stopped by washing with 1xPBS and slides were mounted with Aqua Polymount (PolyScience).

Oil Red O stain

Oil Red O staining was performed on cryo tissue sections. Slides were washed with 60% isopropanol, stained for 10 mins with oil red staining solution (diluted Oil Red O stock solution (0.5 % Oil Red O in 100 % isopropanol) 6:4 in H_2O), washed with 60% isopropanol, counterstained with Mayer's hemalum solution, rinsed with H_20 and mounted.

References

Kishi, S., J. Uchiyama, A. M. Baughman, T. Goto, M. C. Lin and S. B. Tsai (2003). "The zebrafish as a vertebrate model of functional aging and very gradual senescence." Exp Gerontol **38**(7): 777-786.

Leimeister, C., A. Bach and M. Gessler (1998). "Developmental expression patterns of mouse sFRP genes encoding members of the secreted frizzled related protein family." Mech Dev **75**(1-2): 29-42.

Pfaffl, M. W. (2001). "A new mathematical model for relative quantification in real-time RT-PCR." Nucleic Acids Res **29**(9): e45.

Tang, Q., S. Iyer, R. Lobbardi, J. C. Moore, H. Chen, C. Lareau, C. Hebert, M. L. Shaw, C. Neftel, M. L. Suva, C. J. Ceol, A. Bernards, M. Aryee, L. Pinello, I. A. Drummond and D. M. Langenau (2017). "Dissecting hematopoietic and renal cell heterogeneity in adult zebrafish at single-cell resolution using RNA sequencing." J Exp Med **214**(10): 2875-2887.
